# Fly Ash-based Geopolymer Lightweight Concrete Using Foaming Agent

**DOI:** 10.3390/ijms13067186

**Published:** 2012-06-12

**Authors:** Mohd Mustafa Al Bakri Abdullah, Kamarudin Hussin, Mohamed Bnhussain, Khairul Nizar Ismail, Zarina Yahya, Rafiza Abdul Razak

**Affiliations:** 1 Center of Excellence Geopolymer and Green Technology, School of Materials Engineering, Universiti Malaysia Perlis (UniMAP), P.O. Box 77, D/A Pejabat Pos Besar, Kangar Perlis 01000, Malaysia; E-Mails: vc@unimap.edu.my (K.H.); zarinayahya@unimap.edu.my (Z.Y.); rafizarazak@unimap.edu.my (R.A.R.); 2 King Abdul Aziz Science & Technology (KACST), P.O. Box 6086, Riyadh 11442, Kingdom of Saudi Arabia; E-Mail: bnhusain@kacst.edu.sa; 3 School of Environmental Engineering, Universiti Malaysia Perlis (UniMAP), P.O. Box 77, D/A Pejabat Pos Besar, Kangar, Perlis 01000, Malaysia; E-Mail: nizar@unimap.edu.my

**Keywords:** foam concrete, fly ash, geopolymer, alkaline activator, curing temperature

## Abstract

In this paper, we report the results of our investigation on the possibility of producing foam concrete by using a geopolymer system. Class C fly ash was mixed with an alkaline activator solution (a mixture of sodium silicate and NaOH), and foam was added to the geopolymeric mixture to produce lightweight concrete. The NaOH solution was prepared by dilute NaOH pellets with distilled water. The reactives were mixed to produce a homogeneous mixture, which was placed into a 50 mm mold and cured at two different curing temperatures (60 °C and room temperature), for 24 hours. After the curing process, the strengths of the samples were tested on days 1, 7, and 28. The water absorption, porosity, chemical composition, microstructure, XRD and FTIR analyses were studied. The results showed that the sample which was cured at 60 °C (LW2) produced the maximum compressive strength for all tests, (11.03 MPa, 17.59 MPa, and 18.19 MPa) for days 1, 7, and 28, respectively. Also, the water absorption and porosity of LW2 were reduced by 6.78% and 1.22% after 28 days, respectively. The SEM showed that the LW2 sample had a denser matrix than LW1. This was because LW2 was heat cured, which caused the geopolymerization rate to increase, producing a denser matrix. However for LW1, microcracks were present on the surface, which reduced the compressive strength and increased water absorption and porosity.

## 1. Introduction

Lightweight concrete can be prepared either by injecting air or by omitting the finer sizes of the aggregate or by replacing them with hollow, cellular, or porous aggregate. The density of lightweight concrete usually ranges from 300 to 1800 kg/m^3^ [1] whereas the density of normal concrete is approximately 2400 kg/m^3^. Lightweight concrete has been categorized into three groups [2], (1) no-fines concrete; (2) lightweight aggregate concrete; and (3) aerated/foamed concrete. No-fines concrete contains a small amount of aggregate, if any. The coarse aggregate should be a single-size material, with nominal maximum sizes of 10 mm and 20 mm being the most common. The use of blended aggregates (10 and 7 mm; and 20 mm and 14 mm) showed satisfactory performance. However, since this type of concrete is characterized by uniformly distributed voids, it is not suitable for reinforced or pre-stressed concrete used in construction [3]. Lightweight aggregate concrete consists of lightweight aggregate (expanded shale, clay or slate materials that have been fired in a rotary kiln to develop a porous structure) which can be used as a replacement for normal aggregates such as crushed stone or sand [4]. Foamed concrete is produced by using either cement paste or mortar in which large volumes of air are entrapped by using a foaming agent. Such foamed concrete has high flow ability, low weight, and minimal consumption of aggregates, controlled low strength, and excellent thermal-insulation properties [5].

Foamed concrete can be produced either by the pre-foaming method or the mixed-foaming method [1,6]. In the pre-foaming method, a suitable foaming agent is mixed with water, and the foam is combined with paste or mortar. Meanwhile, in the mixed-foaming method, the foaming agent is added to the slurry, and the mixture is whisked into a stable mass that has the required density [1]. The manufacturing of stable mix of foamed concrete depends on many factors, such as the selection of the foaming agent, the method used to prepare the foam to obtain a uniform air-void distribution, selection of materials, strategies for mixture design, and the production of foamed concrete [6]. Various foaming agents have been used to produce foamed concrete, including detergents, resin soap, glue resins, saponin, and hydrolyzed proteins, such as keratin and similar materials [7].

In common foamed concrete, ordinary Portland cement (OPC) and rapid-hardening Portland cement were used [8,9], along with high alumina and calcium sulfoaluminate [10], in order to reduce setting times and improve the early strength. The cost of producing foamed concrete can be reduced by replacing OPC with fly ash [8,10–14] and ground granulated blast-furnace slag [15,16] in quantities of about 30–70% and 10–50%, respectively. With these replacements, the long-term strength of foamed concrete was increased and the heat of hydration was reduced. In addition, the strength of the concrete can be increased by as much as 10% by replacing OPC with silica fume [17–19].

Recently, the potential for replacing the OPC with geopolymer has been explored extensively by researchers. Geopolymer is a term used to describe inorganic polymers based on aluminosilicate, which can be produced by reacting pozzolanic compounds or aluminosilicate source materials with highly alkaline solutions [20]. The aluminosilicate source can be a natural mineral or by-product materials, such as kaolinite, clay, fly ash, silica fume, rice husk ash, or slag. These raw materials must be rich in silicon (Si) and aluminum (Al) in order to produce geopolymer.

Fly ash is suitable for use as a geopolymer source material because it consists mostly of glassy, hollow and spherical particles [21]. Fly ash-based geopolymer cement and concrete have been studied extensively, and they are well known for their properties, which are better than those of normal concrete due to their lower creep [22], lower shrinkage [23], better fire and acid resistance [24], and resistance to sulfate attack [25,26].

However, the manufacturing of fly ash-based geopolymer in terms of lightweight concrete (foamed concrete) has not been explored yet. Hence, the aim of this study was to investigate the properties of fly ash-based foam geopolymer concrete.

## 2. Results and Discussion

### 2.1. X-ray Fluorescence (XRF) Analysis

The comparison of the chemical compositions of the original fly ash and the foamed geopolymer concrete is presented in Table 1. Calcium oxide (CaO) made up 21.6% of the content of the original fly ash, so it must be classified as a class C fly ash (containing more than 20% of CaO) and the ratio of Si:Al was about 3. This fly ash composition is representative of fly ash from the combustion of the sub-bituminous coal that is used in Malaysian power plants [27]. In addition, the content of iron oxide (Fe_2_O_3_) was high, which accounted for the darker color of the fly ash [27]. The powder sample of geopolymers LW1 and LW2 showed increases in the content of SiO_2_. This was due to the reaction between fly ash and the alkaline activator (mixture of sodium silicate and NaOH), which is known as geopolymerization. This process occurs through a mechanism involving the dissolution of the aluminum and silicon species from the surfaces of waste material (fly ash) as well as the surface hydration of undissolved waste particles, followed by the polymerization of active surface groups and soluble species to form a gel and, subsequently, a hardened geopolymer structure [28]. Also, the mass percentages of SiO_2_ and Al_2_O_3_ in LW2 were greater than they were in LW1 due to the heat-induced, rapid geopolymerization process.

### 2.2. Compressive Strength, Density, Porosity and Water Absorption

**Figure 1:**
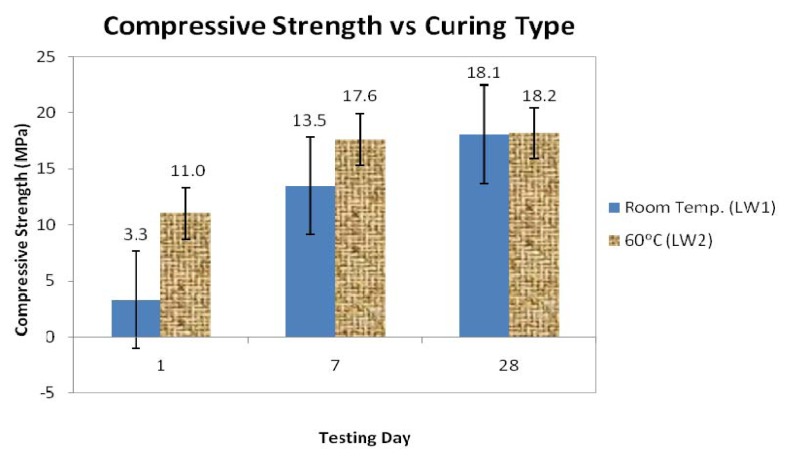
Compressive strengths for two types of foamed geopolymer concrete.

Figure 1 shows the compressive strengths at days 1, 7, and 28 for the foamed geopolymer concretes that were cured at room temperature and at 60 °C for the average of 3 samples. For each of the test days, the maximum compressive strength was observed in the samples that had been cured in the oven (LW2). The maximum compressive strength values for the LW2 samples for days 1, 7, and 28 were 11.0 MPa, 17.6 MPa, and 18.2 MPa, respectively. Thus, we concluded that the curing temperature influenced the strength of the geopolymers [29]. The increase in strength of the LW2 samples was nearly complete after seven days, as evidenced by the fact that the strength had increased only slightly on day 28. However, for LW1, the results showed significant differences in strength for day 1, day 7, and day 28. This proved that heat treatment is required to expedite the rate of development of the strength of the geopolymers.

The average density of LW1 is 1650 kg/m^3^ and for LW2 are 1667 kg/m^3^ as stated in Table 2. The porosity of the foamed geopolymer concrete is the sum of the entrained air voids and the voids within the paste. The higher compressive strength of LW2 samples was due to their lower porosity and water absorption. The LW1 samples had 15.29% porosity and 2.35% water absorption, whereas the LW2 samples had substantially lower corresponding values of 6.78% and 1.22%, respectively, as shown in Table 2. According to BS 1881: Part 122: 1983, low water absorption is deemed to be anything less than 3%, and both types of samples had water absorption values that were less than 3%. Since LW2 is more dense (higher density than LW1), it produced lower porosity and water absorption as mentioned above.

### 2.3. X-ray Diffraction (XRD) Analysis

**Figure 2:**
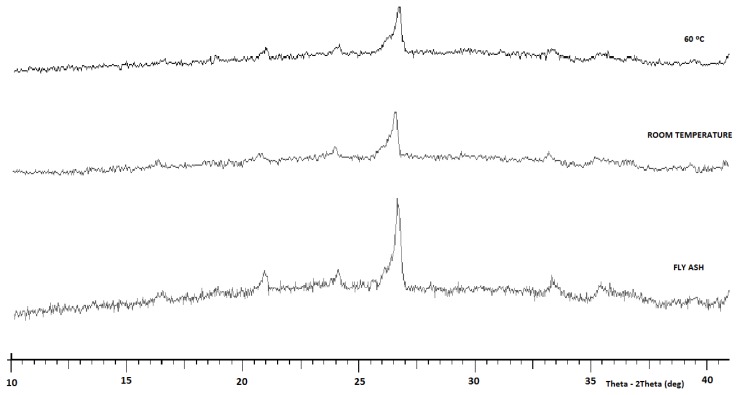
XRD pattern of class C fly ash, foamed geopolymer concretes LW1 (room temperature) and LW2 (60 °C).

The XRD pattern of fly ash was obtained as shown in Figure 2. The main components of the fly ash were quartz, mullite, anhydrite and f-CaO [24]. Figure 2 also shows the foamed geopolymer concrete, which consisted mostly of amorphous content. When comparing the XRD pattern of the original fly ash with the hardened geopolymer, it can be seen that the crystalline phases that existed in the fly ash originally (quartz and mullite) apparently have not been altered by the activation reactions. The fly ash also was made up of an amorphous phase, as indicated by the broad hump registered between 2θ = 20 °C and 30 °C [30].

Additionally, the broad hump between 2θ = 20 °C and 40 °C indicated the characteristic of amorphous gels, including geopolymeric gels and calcium silicate hydrate (C-S-H) gels. This shows that the geopolymeric reaction and the hydrate reaction occurred at the same time [24].

### 2.4. Fourier Transform Infrared Spectroscopy (FTIR) Analysis

**Figure 3:**
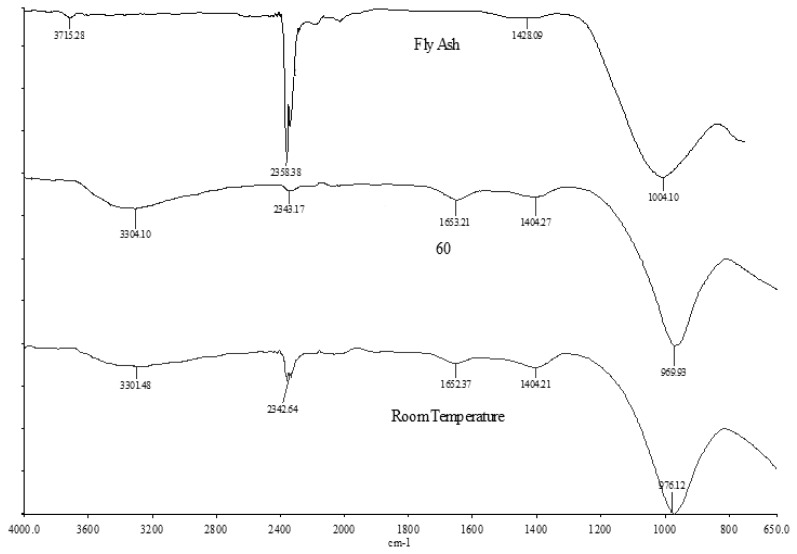
FTIR analysis of fly ash, LW1 and LW2.

Figure 3 shows the IR bands of the fly ash and the foamed geopolymer concrete, and Table 3 summarizes the IR bands obtained from the FTIR analyses. The IR spectrum of fly ash shows main absorption bands at 1004, 1428, 2358, and 3715 cm^−1^. The broad component at 1004 cm^−1^ is due to the Si-O-Si and Al-O-Si asymmetric stretching vibration [31–36] and it becomes sharper and shifts towards lower frequencies (LW1 = 976 cm^−1^ and LW2 = 969 cm^−1^) in lightweight geopolymer. This indicates the formation of a new product (the amorphous aluminosilicate gel phase) due to dissolution of fly in alkaline activator [31–36]. In addition, the band at 1428 cm^−1^ was due to the stretching vibrations of the O-C-O bond indicating the presence of sodium bicarbonate that is suggested to occur due to the atmospheric carbonation of a high alkaline NaOH aqueous phase, which is diffused on the geopolymeric materials surface [31,33,35]. Meanwhile, the broad IR bands at 3715 cm^−1^ and 2358 cm^−1^ represent the stretching and deformation vibration of OH and H-O-H groups, respectively, from the weakly-bound water molecules that were adsorbed on the surface or trapped in the large cavities between the rings of the geopolymeric products [24,37].

Foamed geopolymer samples (LW1 and LW2) showed broad components at 3301 cm^−1^, 2333 cm^−1^, 3304 cm^−1^ and 2343 cm^−1^ which indicated the stretching vibration of OH and H-O-H, respectively [33]. Moreover, bands at 1652 cm^−1^ and 1653 cm^−1^ represent the bending vibration of H-O-H [30].

### 2.5. Microstructure Analysis

**Figure 4:**
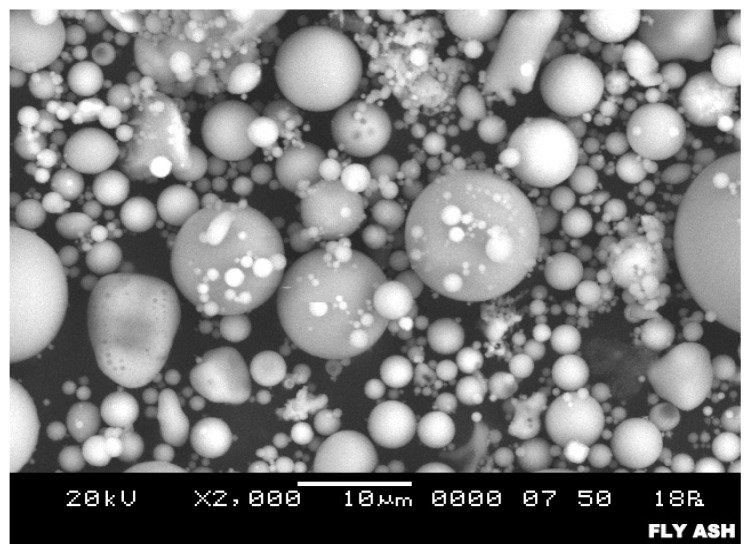
Microstructure of fly ash.

The microstructure of the original fly ash based on the SEM observation is shown in Figure 4. The fly ash consists of spherical, vitreous particles of different sizes. These particles are usually hollow, and some spheres may contain other, smaller particles in their interior [38]. The surface texture of fly ash particles appears to be smooth [39] and also some vitreous, unshaped fragments or quartz particles can be seen [32].

**Figure 5:**
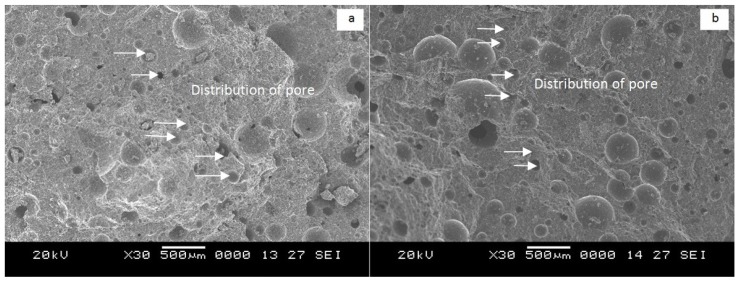

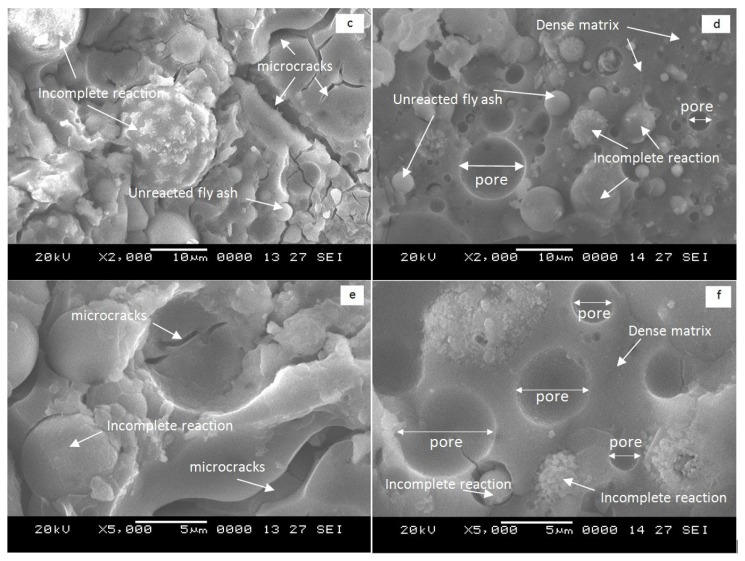
(**a**) Distribution of pores for LW1; (**b**) Distribution of pores for LW2; (**c**) LW1 at a magnification of 2000×; (**d**) LW2 at a magnification of 2000×; (**e**) LW1 at a magnification of 5000×; (**f**) LW2 at a magnification of 5000×.

Figure 5a–d shows the foamed geopolymer concrete of LW1 and LW2 at different magnifications. The size of the pores in the foamed geopolymer concrete ranged from 4 μm to 37 μm, and the distributions of the pores in both samples were uniform, as shown in Figure 5a,b. As expected, the existence of these pores in the foamed concrete result in its being classified as lightweight concrete. However, at magnifications of 2000× and 5000×, microcracks were observed in the LW1 samples (Figure 5c,e), which contributed to their lower strength by increasing their water absorption and porosity.

The LW2 samples, which had a denser matrix (Figure 5d,f) than the LW1 samples, produced foamed geopolymer concrete that had greater strength. These stronger samples were heat cured, which facilitated the complete reaction between the fly ash and the alkaline activator to form aluminosilicate gel. Soon after the mixing process, the gel covered the fly ash particles and produced a dense matrix (complete reaction). Nevertheless, there were still some instances of incomplete reaction, as evidenced by the fact that the surface of the fly ash was covered with aluminosilicate gel rather a dense matrix having been formed. This situation was observed on both samples.

Unreacted fly ash was present in both samples. Fly ash with its original spherical shape (Figure 4) was located on the nearby dense matrix. From the SEM analysis, it was determined that the existence of microcracks and the incomplete formation of the dense matrix had caused water absorption and porosity of the LW1 samples to increase, thereby impairing their strength.

## 3. Experimental Section

### 3.1. Materials

Fly ash, sodium silicate, sodium hydroxide (NaOH) and foaming agent (superplasticizer) were used to produce the foam geopolymer concrete. The fly ash was obtained from Manjung Power Station in Lumut, Perak, Malaysia. The chemical composition of the fly ash was determined by X-ray Fluorescence (XRF) as shown in Table 1. The microstructure of the fly ash is shown in Figure 4.

Sodium silicate and the NaOH solution were mixed together to act as the activator. NaOH pellets with 99% purity, made in Taiwan with the brand name of Formosoda-P were used to produce 12 M NaOH solution by adding the NaOH pellets to distilled water. This concentration was based on previous research [40] that indicated that the maximum strength of geopolymer was obtained when 12 M NaOH was used. Meanwhile, a technical grade of sodium silicate was obtained from South Pacific Chemical Industries Sdn. Bhd. (SPCI), Malaysia, with a chemical composition of SiO_2_ = 30.1%, Na_2_O = 9.4%, and H_2_O = 60.5% (SiO_2_/Na_2_O = 3.2). The other characteristics were: specific gravity at 20 °C = 1.4 kg/cm^3^ and viscosity = 0.4 Pa s.

### 3.2. Mix Design and Mixing Process

In order to produce a desirable strength in lightweight concrete, a trial and error process was commonly used [41]. Since no methods have been proposed for producing foamed geopolymer concrete, we decided to use a geopolymer paste-to-foam ratio of 1:2 (by volume). The foam geopolymer concrete was produced by using the pre-foaming method, in which the foam is produced separately and then mixed with the geopolymer paste. The foam was produced by diluting the foaming agent with water based on a foaming agent-to-water ratio of 1:20 by volume. Then, the foam was generated by using a custom-made foam-generating machine: LCM Model 02. The geopolymer paste was produced according to the ratios shown in Table 4.

The sodium silicate and NaOH were mixed together for three minutes and then mixed with fly ash for another five minutes. After the geopolymer paste was homogeneous, the foam was added and mixed for another five minutes before it was placed in the 50-mm mold. The samples were cured at room temperature (LW1) and 60 °C (LW2). The LW2 samples were cured in the oven for 24 hours and then left at room temperature (open air) until compressive strength testing were conducted. Meanwhile, LW1 was cured at room temperature (open air) until testing day.

### 3.3. Testing

#### 3.3.1. Compressive Strength

Compressive strength test of all samples were evaluated according to ASTM C 109/C 109 M by using the Shimadzu Universal Testing Machine. A minimum of three samples was tested to evaluate the compressive strength. The samples were tested on days 1, 7, and 28.

#### 3.3.2. The Water Absorption

The water absorption was determined according to ASTM C642 and was calculated by the equation (Equation (1)):

(1) $$\text{Water absorption}=[({\text{M}}_{\text{s}}-{\text{M}}_{\text{d}})/{\text{M}}_{\text{d}}]\times 100$$

M_s_ = mass of surface-dried sample (g); M_d_ = mass of oven-dried sample (g).

#### 3.3.3. Porosity

The porosity was determined according to ASTM C642 and was calculated by the equation (Equation 2):

(2) $$\text{Porosity}=[({\text{M}}_{\text{w}}-{\text{M}}_{\text{d}})/{\text{M}}_{\text{w}}-{\text{M}}_{\text{s}}]\times 100$$

M_w_ = mass of specimen after immersion in water (g); M_d_ = mass of specimen after oven dried (g); M_s_ = mass of specimen suspended in water (g).

#### 3.3.4. X-ray Diffraction (XRD)

The samples were prepared in powder form and analyzed with XRD to determine the pattern of the crystalline phase. XRD analysis was conducted using XRD–6000, Shimadzu X-ray diffractometer with Cu Kα radiation and with auto-search/match software as standard to aid qualitative analysis.

#### 3.3.5. Scanning Electron Microscope (SEM)

The microstructure of the foamed geopolymer concretes with different curing temperatures was determined with a JSM-6460LA model Scanning Electron Microscope (JEOL). The specimens were cut into small pieces before observation.

#### 3.3.6. Fourier Transform Infrared Spectroscopy (FTIR)

Using samples in powder form, infrared bands were recorded for wavelengths between 4000 cm^−1^ to 650 cm^−1^ using a Perkin Elmer FTIR Spectrum RX1 Spectrometer. The specimen for testing was prepared using the KBr pellet technique. Potassium Bromide (KBr) and sample powders were put into a mold and compressed by using cold press machine for 2 minutes at a load of 4 tons.

## 4. Conclusions

The results of the experimental study led to the following conclusions:

- The compressive strength of foamed geopolymer concrete LW2 with heat curing (60 °C) produced the maximum compressive strength on days 1, 7, and 28 (11.0, 17.6, and 18.2 MPa), respectively.
- The compressive strength of the LW2 samples was greater than the compressive strength of the LW1 samples. This was attributed to the fact that the porosity and water absorption of the LW2 samples, at 6.78% and 1.22% respectively, were lower than the porosity and water absorption of the LW1 samples, at 15.29% and 2.35%, respectively.
- Based on SEM observations, the LW2 samples had a denser matrix than the LW1 samples. This occurred because heat curing increased the rate of geopolymerization and hence, increased the strength. The LW1 samples had microcracks that resulted in increased water absorption and porosity, thus the strength was reduced.

## Figures and Tables

**Table 1:** Composition of fly ash and foam geopolymer concrete as determined by XRF analysis (mass %).

Chemical Composition	Fly Ash	Samples Cured at Room Temperature (LW1)	Samples Cured at 60 °C (LW2)
SiO_2_	26.4	35.1	37.6
Al_2_O_3_	9.3	11.8	12.8
CaO	21.6	19.6	18.7
Fe_2_O_3_	30.1	23.3	21.6
MnO	0.3	0.2	0.2
TiO_2_	3.1	2.3	2.10
K_2_O	2.6	2.7	2.7
SO_3_	1.3	0.9	0.8

**Table 2:** Density, porosity and water absorption of foamed geopolymer concretes.

Sample	Curing	Compressive Strength (Mpa)			Porosity (%)	Water Absorption (%)	Density (kg/m^3^)

		Day 1	Day 7	Day 28			
LW1	Room temp.	3.3	13.5	18.1	15.29	2.35	1650
LW2	60 °C	11.0	17.6	18.2	6.78	1.22	1667

**Table 3:** Characteristic of IR band for foamed geopolymer concrete.

Bonds	Fly Ash (cm^−1^)	LW1 (cm^−1^)	LW2 (cm^−1^)
Stretching vibration (OH, H-O-H) [24,33,37]	3715–2358	3301–2333	3304–2343
Bending vibration (H-O-H) [30]	-	1652	1653
Stretching vibration (O-C-O) [31,33,35]	1437	-	-
Asymmetric stretching (Si-O-Si & Al-O-Si) [31–36]	1082	970	969

**Table 4:** Mix design for foam geopolymer concrete.

Sample	Fly Ash: Activator	Sodium Silicate: NaOH (Activator)	Foam: Geopolymer Paste	Curing Temperature
LW1	2:1	2.5:1	2:1	Room temperature
LW2	2:1	2.5:1	2:1	60°C
